# Integrated neurobehavioral and organ-specific safety profiling of baicalin: acute/subacute toxicity studies

**DOI:** 10.3389/fphar.2025.1607919

**Published:** 2025-08-21

**Authors:** Jiali Yang, Mengyu Chen, Wan Zhang, Jia Liu, Jing Zhao, Xin Ping, Ye Lu, Pei He, Lin Pei

**Affiliations:** ^1^School of Integrated Traditional Chinese and Western Medicine, Hebei University of Chinese Medicine, Shijiazhuang, Hebei, China; ^2^Key Research Laboratory of Phlegm Stagnation Syndrome and Treatment in Hebei Province, Hebei Academy of Chinese Medicine Sciences, Shijiazhuang, Hebei, China; ^3^ The Fourth Affiliated Hospital of Hebei University of Chinese Medicine, Shijiazhuang, Hebei, China; ^4^ The First Affiliated Hospital of Hebei University of Chinese Medicine, Shijiazhuang, Hebei, China; ^5^Internal Medicine Department, Hospital of Renmin University of China, Beijing, China

**Keywords:** baicalin, acute toxicity, subacute toxicity, behavioral tests, histopathology, hematology abstract

## Abstract

**Ethnopharmacological relevance:**

Baicalin, an extract derived from the dried root of Scutellaria baicalensis Georgi (Huang Qin), has demonstrated neuroprotective properties. Nonetheless, the safety profile of baicalin has not yet been fully elucidated.

**Aim of the study:**

The objective was to characterize the acute and subacute toxicity profiles of baicalin across various organ systems, thereby establishing safe therapeutic windows for its clinical application in the treatment of chronic neurodegenerative disorders.

**Materials and methods:**

Acute toxicity was assessed at 4,000 mg/kg (OECD 423), while subacute toxicity evaluated escalating doses (1,000–4,000 mg/kg; OECD 407). Endpoints included survival, general behaviours, behavioral alterations, hematological/biochemical parameters, organ coefficients, and histopathology of brain, liver, and kidney.

**Results:**

Acute exposure showed no mortality (LD50 > 4,000 mg/kg) or lasting physiological effects, with only transient gastrointestinal symptoms in one subject. Subacute administration caused temporary gastrointestinal issues and occasional compulsive behaviors, all resolving within 24 h. Behavioral assessments indicated intact neurocognitive function and emotional stability. Hematological profiles revealed sex-specific responses, with males showing higher lymphocyte percentages and females demonstrating renal changes. Biochemical analyses indicated liver metabolic changes, including alkaline phosphatase suppression and reduced triglycerides, along with mild nephrotoxic signs. Histopathological evaluations confirmed non-necrotic liver stress and unchanged hippocampal structure.

**Conclusion:**

Baicalin showed high acute safety with an LD50 over 4,000 mg/kg in mice, and a subacute no-observed-adverse-effect level (NOAEL) of 2,000 mg/kg, indicating its potential as a neuroprotective agent. However, 4,000 mg/kg doses led to reversible hepatorenal toxicity and biochemical alterations, highlighting the need to monitor organ function during extended high-dose use.

## Graphical Abstract

**Figure:**
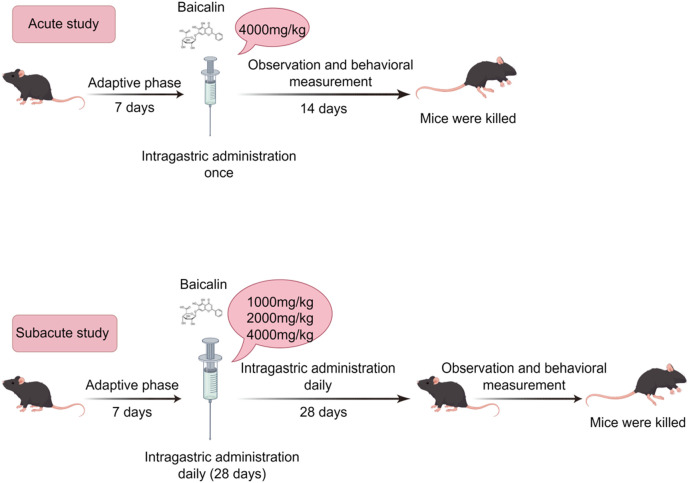
For acute toxicity assessment, C57BL/6 mice underwent a 7-day acclimation period prior to receiving a single oral gavage dose of 4,000 mg/kg baicalin. Behavioral evaluations were performed for 14 days post-administration. In subacute toxicity studies, different doses of baicalin were administered via daily oral gavage for 28 consecutive days, supplemented by daily behavioral assessments and behavioral testing protocols.

## 1 Introduction

**FIGURE 1:**
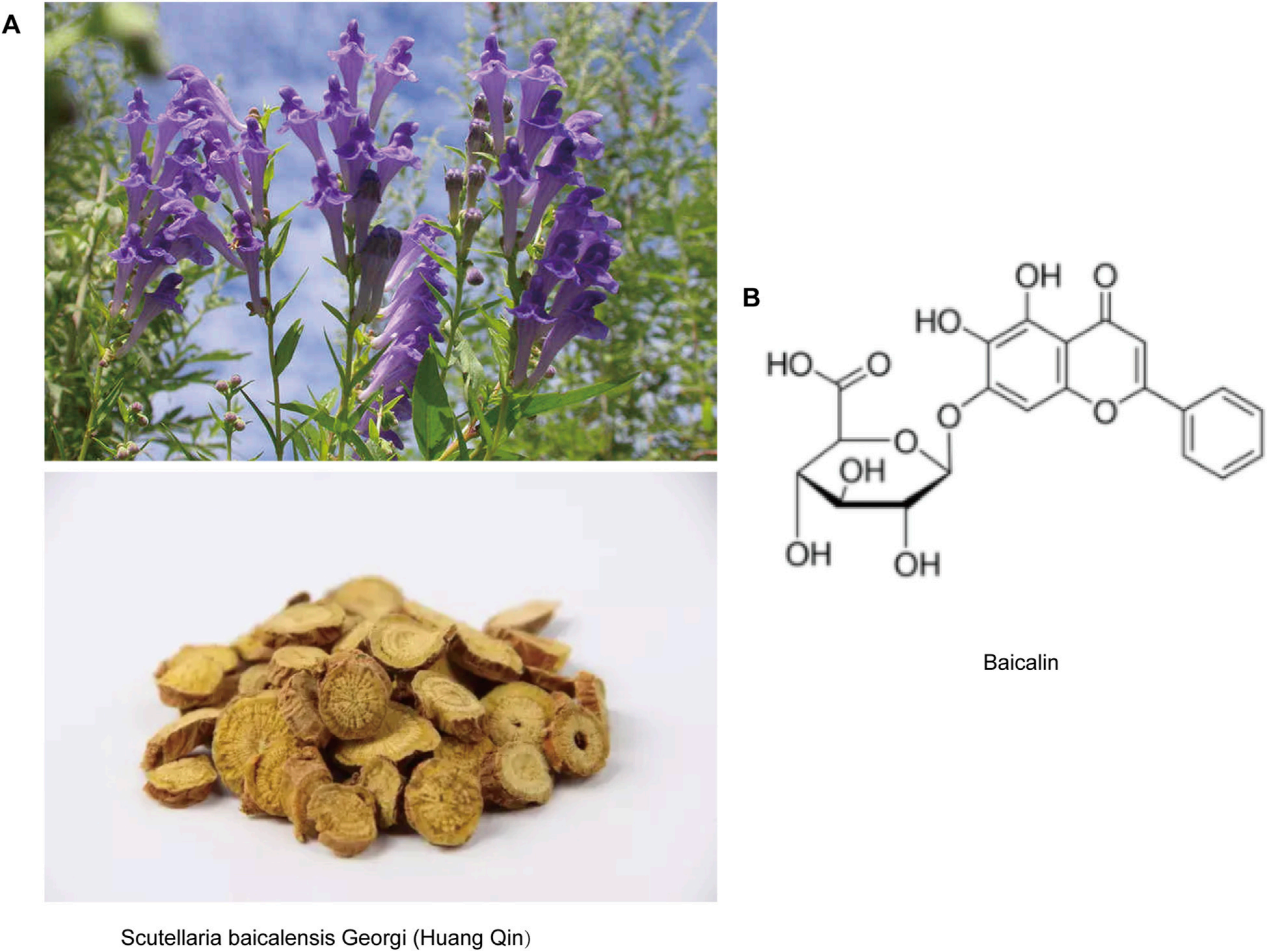
Representative image of baicalin. **(A)** Pictures of Scutellaria baicalensis Georgi (Huang-Qin) plants and herbal tablets. **(B)** Molecular structure of baicalin.

Scutellaria baicalensis Georgi (Huang-Qin), a important botanical agent in traditional Chinese medicine with a pharmacopeial history dating back to the Shennong Bencao Jing (Divine Farmer’s Materia Medica, c. 200–250 CE), has been systematically documented for its heat-clearing, detoxifying, and pregnancy-stabilizing properties (Wang et al., 2018; Li et al., 2020). Modern pharmacological studies have shown that Huang Qin has anti-inflammatory, antioxidant, neuroprotective and immune-enhancing effects. Baicalin (C21H18O11; 7-d-glucuronic acid-5,6-dihydroxyflavone) (Figure 1), an extract from the dried root of Huang Qin, has been demonstrated multi-target pharmacological efficacy against viral infections, oxidative stress, tumorigenesis, and inflammatory processes (Hu et al., 2022; Hu et al., 2023). Notably, its neuroprotective properties have gained particular therapeutic significance, as evidenced by preclinical studies showing that baicalin significantly reduces levels of tumor necrosis factor-α (TNF-α) and interleukin-6 (IL-6) while inhibiting microglial overactivation in the model of cerebral ischemia (Yang S. et al., 2019; Wang H. et al., 2024; Li et al., 2025). Similarly, in a mouse model of depression, a 60 mg/kg dose alleviates hippocampal inflammatory damage (Guo et al., 2019). These findings not only provide scientific validation for the herb’s ancient applications in brain disorder treatments but also position Huang-Qin as a promising candidate for developing next-generation neurotherapeutics targeting neurodegeneration, effectively bridging ancient herbal knowledge with modern neuroscience paradigms.

Despite extensive investigations into neuroprotective mechanisms of baicalin, its toxicological profile, particularly under high-dose or prolonged exposure scenarios, remains poorly characterized. Existing studies primarily focus on efficacy at low to moderate doses (50–400 mg/kg) (Xi et al., 2016; Zhang et al., 2017; Si et al., 2025), overlooking the cumulative organ-specific toxicity and neurobehavioral sequelae that may emerge during chronic administration—a critical knowledge gap given its proposed clinical use in long-term neurodegenerative therapies. Notably, clinical data shows no signs of hepatic or renal toxicity when assesses with a single oral dose of baicalein in high doses (Li et al., 2014; Si et al., 2025). This knowledge gap is concerning, as structurally analogous flavonoids (e.g., quercetin and silymarin) have demonstrated hepatotoxicity and nephrotoxicity in both preclinical and clinical settings (Harwood et al., 2007; Singh and Semwal, 2024).

Comprehensive toxicity evaluation is an indispensable step in drug development, bridging the gap between therapeutic potential and clinical translatability (Yang et al., 2024). For natural products like baicalin, which are often perceived as inherently safe, but rigorous safety assessments are imperative to define therapeutic windows and mitigate off-target organ damage. Critical questions remain unanswered: What is the maximum tolerated dose of baicalin in acute exposure? Which organs are vulnerable to subacute toxicity? Given its putative neuroprotective properties, might chronic high-dose baicalin exposure paradoxically evoke neurotoxic responses?

In this study, we integrated behavioral and toxicological approaches to conduct a comprehensive analysis of the acute (14-day) and sub-acute (28-day) toxicity of baicalin in C57BL/6J mice for the first time. We simultaneously evaluated: (1) survival, body weight, and general behavior; (2) anxiety/depression-like behaviors and motor function; (3) hematological, hepatic, and renal parameters; (4) histopathology of brain, liver, and kidney. This systematic framework not only identifies baicalin’s toxicity thresholds but also establishes correlations between behavioral deficits and organ-specific damage, providing a blueprint for natural product safety evaluation.

## 2 Materials and methods

### 2.1 Animals

A total of sixty 8-week-old C57BL/6J mice (30 males and 30 females) were procured from Beijing Vital River Laboratory Animal Technology Co., Ltd. (License No. SCXK [Jing] 2021-0006). All animals were housed in the Animal Center of the Fourth Affiliated Hospital of Hebei University of Chinese Medicine for 1 week, and then the follow-up experiments were carried out (22°C ± 1°C, 50% ± 10% humidity, 12-h light/dark cycle). This study was approved by the Ethics Committee of Hebei University of Chinese Medicine (Ethics Approval No. DWLL202403137).

### 2.2 Acute oral toxicity study

The 4,000 mg/kg dose of baicalin (Shanghai Yuanye Biotechnology Co. B20570, ≥98% purity) was selected in acute oral toxicity study. The acute toxicity assessment was conducted in strict compliance with OECD Guideline 423 (Acute Oral Toxicity–Acute Toxic Class Method) (Liu et al., 2024). A limit test dose of 4,000 mg/kg was selected based on: (1) Preliminary evidence indicates that baicalin, which is formed by the conjugation of the C7 hydroxyl group of baicalein with glucuronic acid, is likely to possess low acute toxicity based on its structural analogy to low-toxicity analogs such as baicalein (Zheng et al., 2017; Dai et al., 2018); (2) Equivalent dose calculations were performed by converting baicalein and baicalin, with adjustments for species-specific metabolic differences and bioavailability variations (Table 1) (Dong et al., 2021); (3) No adverse effects were observed in the subacute toxicity study (e.g., 500 mg/kg in beagle dogs with UP446 formulation containing 60% baicalin) (Yimam et al., 2016); (4) Baicalin is suitable for oral administration but has low bioavailability (Ganguly et al., 2022).

**TABLE 1:** Dose comparison between baicalin and baicalein.

Parameter	Mouse study (baicalin)	Human study (baicalein)
Single-dose	4,000 mg/kg	2,800 mg (∼46.7 mg/kg)
Subacute dose	1,000–4,000 mg/kg/day ×28 days	Not applicable
Bioavailability	2%–5% (oral)	20%–30% (oral)
Metabolic Pathway	Hydrolysis to baicalein	Direct absorption

Twenty C57BL/6J mice (10 males and 10 females) were stratified by sex and randomly assigned to two experimental groups (*n* = 10 per group; five males and five females) through block randomization: Control group was administered a single oral gavage of sterile saline (0.9% NaCl, 20 mL/kg body weight). Baicalin group was received a single high-dose oral suspension of baicalin (4,000 mg/kg in saline, equivalent volume of 20 mL/kg).

In the experimental protocol, animals underwent a 12-h fasting period with water provided to standardize their metabolic status. To minimize circadian variability, baicalin or sterile saline was administered as a single dose at 8:00–10:00 a.m. Post-administration, 24-h temporary observation period was implemented with evaluations at 0.5, 1, 2, 4, 8, and 24 h focusing on survival, neurological signs (e.g., convulsions, ataxia), and autonomic reactions (e.g., lacrimation, piloerection). Extending beyond the critical phase, a comprehensive observation period from Day 2 to Day 14 involved assessments of mortality, body weight and food intake, as well as the manifestation of clinical symptoms like diarrhea and lethargy. All items to be observed were referred to in Table 2 to ensure continuity and completeness of experimental observations (Ru et al., 2022; Wen et al., 2023). Following the single gavage administration, a comprehensive battery of behavioral assessments was conducted.

**TABLE 2:** Animal toxicity observation table.

Parameters	Content	Time
General observations	A. Changes in body surface hair, such as piloerection, alopecia, and bald patches	Observation period
	B. Skin rupture, redness, swelling, and scarring	
	C. Changes in body temperature, including hypothermia or hyperthermia	
	D. Tearing or bloody tears, mydriasis or miosis, exophthalmos, ptosis or blepharoptosis	
Motor function	A. Spontaneous activity, grooming behavior, and frequency of movement	
	B. Drowsiness and ease of arousal	
	C. Motor dysfunction, spasms, tremors, hyperactivity, depression, or immobility	
	D. Involuntary contractions of limb muscles	
	E. Convulsive contractions, clonic convulsions, and syncope	
Respiratory system	A. Dyspnea, wheezing, and decreased respiratory rate	
	B. Shortness of breath or respiratory pauses	
	C. Abdominal retraction during inhalation	
	D. Changes in nasal secretions	
Stimulus response	A. Alterations in the reflex ability to external stimuli (such as turning, touch, and noise)	
	B. Reduced responsiveness to pain stimuli or analgesia	
Secretions and excretions	A. Hypersalivation, vomiting, or retching	
	B. Dry or watery feces	
	C. Hematuria, urinary incontinence	
Weight	Recorded changes in body weight	
Food intake	Recorded food consumption	
Organ index	Organ weight/body weight	Mice were dissected
Pathology	Dissect all mice in the experiment. Record changes in the position, color, and size of organs, and conduct histopathological examinations of the liver and kidneys	
Blood tests	Hematological and biochemical tests	
Mortality	Observe the onset time, severity, and duration of toxic symptoms. If a mouse dies, record the time of death, associated reactions, and explore the cause	Everyday

### 2.3 Subacute toxicity study

In accordance with OECD Guideline 407 for Repeated Dose Oral Toxicity Studies in Rodents, a 28-day subacute toxicity assessment was conducted with rigorous attention to experimental design and group (Ramesh and Mandal, 2019). Forty C57BL/6J mice (20 males and 20 females), were randomized into four groups (*n* = 10 per group, with a sex ratio of five males to five females). The C57BL/6J mouse strain was selected owing to its genetic uniformity, adherence to OECD toxicity testing guidelines, and comprehensive pathological characterization within toxicological research (Yang et al., 2022; Corder et al., 2023). The groups were administered as follows: the Normal group received the vehicle (0.9% NaCl, 20 mL/kg body weight), while the low-dose, mid-dose, and high-dose groups were given 1,000 mg/kg, 2,000 mg/kg, and 4,000 mg/kg of baicalin, respectively. The selection of the dose range was based on acute toxicity findings, which indicated no mortality at 4,000 mg/kg.

Baicalin and NaCl were administered via oral gavage at a volume of 20 mL/kg between 08:00 and 10:00 a.m. for 28 consecutive days. Clinical monitoring included daily recordings of body weight and food intake. Additionally, assessment of general status, including observations of abnormal behaviors (such as hyperactivity and circling), and mortality, were scored according to Table 2. At the end of gavage, a series of behavioral experiments were performed.

At the end of the study, mice were deeply anesthetized with 5% isoflurane for induction, followed by maintenance at 2% (RWD Life Science). Cardiac puncture was conducted to collect whole blood for hematological and biochemical analyses. Necropsy was then carried out, the major organs (liver, kidneys, brain, spleen, and heart) were excised (weighed to the nearest 0.01 g). This comprehensive experimental design ensured the accuracy and reliability of the subacute toxicity assessment of baicalin in mice.

### 2.4 Behavioral testing

#### 2.4.1 The sucrose preference test (SPT)

Anhedonia was evaluated using the validated sucrose preference test. During the 48-h habituation phase, mice had free access to both 1% sucrose solution and tap water, with bottle positions alternated every 24 h to minimize side bias. 12 h before the start of the official experiment, animals underwent food deprivation while maintaining free water access. The consumption of aqueous sucrose solution and purified water was then measured over a 16-h period, with the bottle position rotated every 8 h to account for positional preference. The sucrose preference rate was calculated as (Sucrose water consumption/(Sucrose water consumption + Pure water consumption)×100%) (Zhu et al., 2024).

#### 2.4.2 The tail suspension test (TST)

TST had been tested to determine depressive-like moods. Mice were suspended by their tails, which were fixed to an iron frame 50 cm above the ground. The test lasted for 6 min, during which the mice were allowed to adapt for 2 min before the time and number of struggles were recorded. After the test, the feces on the ground were removed, and the area was wiped with 75% alcohol to eliminate any odors (Li X. et al., 2018).

#### 2.4.3 The forced swim test (FST)

Despair-like emotions were assessed using the FST. Mice were placed in a vertical transparent plastic cylinder (diameter 30 cm, height 50 cm, water depth 30 cm), and the water temperature will be maintained at 25°C (Agostinho et al., 2019). The test lasted for 6 min, during which the mice were allowed to acclimate for 2 min before the struggle time and frequency (defined as significant movement of all four limbs) were recorded. After each experiment, the water was changed to ensure there were no residual odors (Katrancha et al., 2019).

#### 2.4.4 The marble-burying test (MBT)

Anxiety - like behaviors were assessed using MBT. Twenty clean standard glass marbles (various styles and colors, 15 mm in diameter, weighing approximately 5.2 g) were gently placed on the surface of the bedding material. Mice were placed in one corner of the cage containing the marbles and carefully positioned in the marble - free area of the test cage facing the marbles. After the test, the mice were removed and returned to their home cages. Scoring was performed blind to the treatment conditions or genotypes of the tested mice, with two to three scorers counting the number of buried marbles. A marble was considered buried if at least two-thirds (or sometimes three-quarters) of its surface area was covered by the bedding material (Borgonetti et al., 2020).

#### 2.4.5 The pole test

The pole test is a behavioral test widely used for assessing motor dysfunction in mice. This procedure requires mice to grip and manipulate themselves down to the bottom of the pole, which can evaluate the animals’ motor coordination abilities. During the test, the mouse was placed at the top of the pole with its head facing upwards, and the animal will naturally orient itself downward and descend along the pole to the ground without interruption to return to its home cage. The total time taken for the mouse to descend to the bottom of the pole was recorded (Yu et al., 2020).

#### 2.4.6 The sucrose splash test (SST)

SST is a method used to assess the motivational and self-care behaviors of rodents. In this test, a 10% sucrose solution was sprayed onto the dorsal surface of the rodents’ fur. The grooming behavior, which was triggered by the rodents to remove the solution through licking, biting, or scratching, was then measured. The frequency and duration of grooming behavior were recorded within 5 min after the evaporation of the sucrose solution (Jiang et al., 2019).

#### 2.4.7 The novel object recognition test (NOR)

Exploiting rodents’ innate preference for novel object exploration, NOR is utilized to assess murine learning and memory. The protocol involved three phases: acclimation, training, and testing. During acclimation, mice explored an arena freely for 5 min. In training, two identical objects (A and B) were placed in opposite corners, and mice explored for 5 min. After 24 h, object A was replaced with a new cube (C) for testing, and mice explored again for 5 min. Exploration was defined as the snout being oriented toward or contacting the object within 2 cm. The discrimination index (DI) was calculated as DI (%) = (Time exploring C (TC)/(Time exploring B (TB) + Time exploring C (TC))) × 100%. This protocol effectively measures recognition memory in mice by controlling extraneous variables.

#### 2.4.8 The nest shredding test (NST)

The nest - building assay was conducted by housing mice individually in transparent polycarbonate cages (33 cm × 18 cm × 15 cm) containing 1 cm of autoclaved bedding material. A pre-weighed sterile cotton fiber nestlet (12.7 cm × 12.7 cm, 1.3 cm thickness) was positioned centrally on the bedding surface. After a 2-h acclimation period without disturbance, the remaining intact nest material was carefully collected and reweighed. The percentage of nest material shredded by each mouse was calculated using the formula: [(initial weight–final weight)/initial weight] × 100%. This methodologically standardized protocol allowed for objective quantification of nest-building behavior as a measure of motivational drive and affective state (Martinez Hernandez et al., 2021).

All behavioral assessments were performed by trained investigators blinded to treatment groups. To minimize stress accumulation, tests were scheduled with ≥24-h intervals in a randomized order, and environmental variables (noise, lighting) were strictly controlled. Each apparatus was cleaned with ethanol between trials to eliminate olfactory cues.

### 2.5 Analysis of hematological and biochemical indices

To obtain blood samples, an anticoagulant-containing vessel was utilized to collect approximately 0.5 mL of blood, which was promptly processed using a state-of-the-art fully automated hematological analyzer (Mindray BC6800 plus, Shenzhen, China). This analysis facilitated the determination of various hematological indices, encompassing the white blood cell count (WBC), the percentages of neutrophils (NEU%), the percentages of lymphocytes (LYM%), the percentages of monocytes (MON%), Eosinophil (EOS), basophil (BASO), red blood cell count (RBC), hemoglobin levels (HGB) and hematocrit value (HCT).

Additionally, approximately 1.8 mL of blood were aspirated and subjected to centrifugation at 3,000 revolutions per minute for 10 min, yielding serum for the subsequent assessment of enzymatic activities employing an advanced analyzer (Rayto Life Technologies, Chemray 800, Shenzhen, China). This evaluation encompassed the measurement of serum alanine aminotransferase (ALT), aspartate aminotransferase (AST), alkaline phosphatase (ALP), uric acid (UA), serum urea nitrogen (BUN), blood creatinine (Scr), total cholesterol (TC), triglycerides (TG), albumin (ALB), total protein (TP) and globulin (Glob).

### 2.6 Gross anatomy and histopathologic examination

Organ weights were immediately recorded using a precision analytical balance (±0.001 g) to minimize *postmortem* hydration changes. All measurements were performed by two independent investigators to ensure reproducibility. Two standardized indices were derived to assess dose-dependent organotoxicity: Organ-to-Body Weight Ratio (OBR%) = (Organ Wet Weight (g)/Total Body Weight (g))×100%. Organ-to-Brain Weight Ratio (OBBR%) = (Organ Wet Weight (g)/Brain Wet Weight (g))×100%. OBR normalizes organ mass to whole-body growth, identifying disproportionate organ enlargement/atrophy. OBBR controls for inter-individual neurodevelopmental variability, isolating organ-specific toxicity.

### 2.7 Hematoxylin-eosin (HE) staining procedure

The sections were dewaxed in xylene for 20 min, followed by sequential hydration in 100%, 95%, 80%, and 75% ethanol for 5 min each. Afterward, the sections were stained with hematoxylin for 5 min, differentiated in hydrochloric acid-ethanol for 30 s, and washed in tap water for 10 min to allow for bluing. Subsequently, the sections were stained with eosin for 3 min, dehydrated through a graded ethanol series, cleared in xylene, and mounted with neutral resin. Finally, the sections were observed under a microscope.

### 2.8 Statistical analysis

The statistical analysis was performed utilizing GraphPad Prism version and SPSS, with graphical representations created using Adobe Illustrator. For acute toxicity endpoints, intergroup comparisons between control and baicalin group were performed using two-tailed unpaired Student's t-tests following verification of normality (Shapiro-Wilk test) and homogeneity of variance (Levene’s test). Subacute toxicity datasets involving multiple dose groups were analyzed via one-way analysis of variance (ANOVA) with Tukey’s *post hoc* test. Data were expressed as the mean ± SEM. Statistical significance was determined at a threshold of *P* < 0.05.

## 3 Results

### 3.1 General behaviors of acute oral toxicity in mice

**FIGURE 2:**
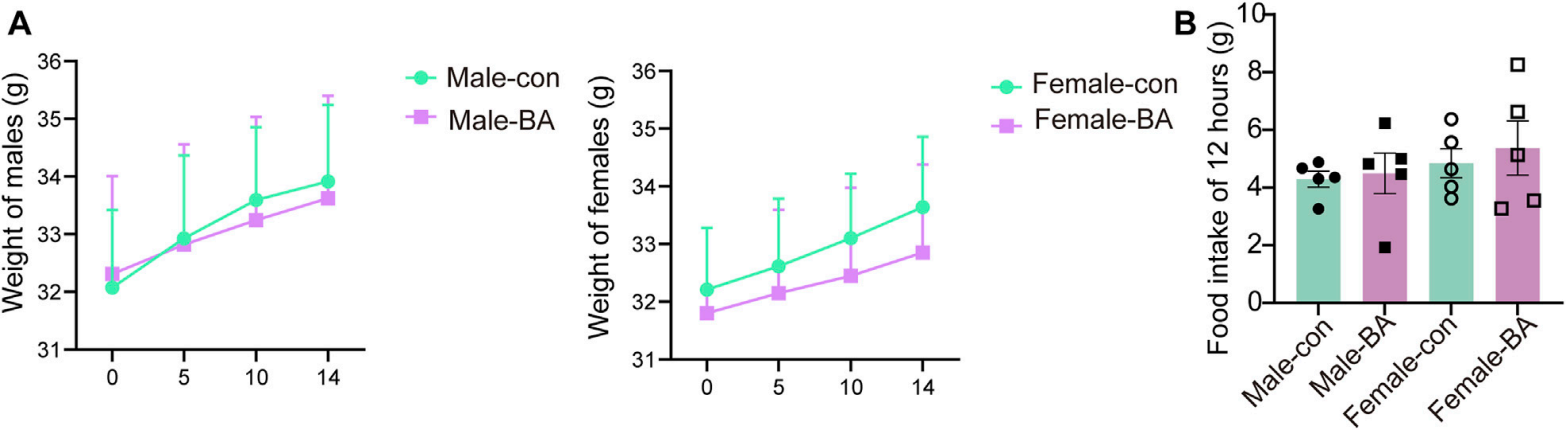
Weight and diet in acute toxicology experiments. **(A)** Weight gain of male and female mice over 14 days. **(B)** Food intake of 12 h. Values were expressed as the mean ± SEM (*n* = 5).

All subjects survived with no treatment-related deaths in the 14-day trial (detailed in Table 3). One male in the baicalin group was observed to have loose stools after gavage, but it disappeared after 24 h. Daily surveillance revealed no detectable pathophysiological alterations in other mice. Longitudinal monitoring showed progressive body weight gain trajectories in all groups, consistent with normal mice growth patterns (Figure 2A, *P* > 0.05). The results confirmed the absence of significant treatment effects on weekly body mass fluctuations. For 12 h of food intake, there was no significant difference between the groups (Figure 2B, *P* > 0.05). According to OECD Guideline 420 criteria, the calculated median lethal dose (LD50) of baicalin exceeded 4,000 mg/kg, confirming its favorable acute safety profile even at supratherapeutic doses tested (Nascimento et al., 2020).

**TABLE 3:** General behavior of acute toxicity studies.

Parameters	Control group (0.9% NaCl)		Baicalin group (4,000 mg/kg)	
	♂ (*n* = 5)	♀ (*n* = 5)	♂ (*n* = 5)	♀ (*n* = 5)
Appearance signs	0/5	0/5	0/5	0/5
Behavioral activities	0/5	0/5	0/5	0/5
Stimulus response	0/5	0/5	0/5	0/5
Secretions/excretions	0/5	0/5	1/5	0/5
Death	0/5	0/5	0/5	0/5

### 3.2 General behaviors of subacute oral toxicity in mice

**FIGURE 3:**
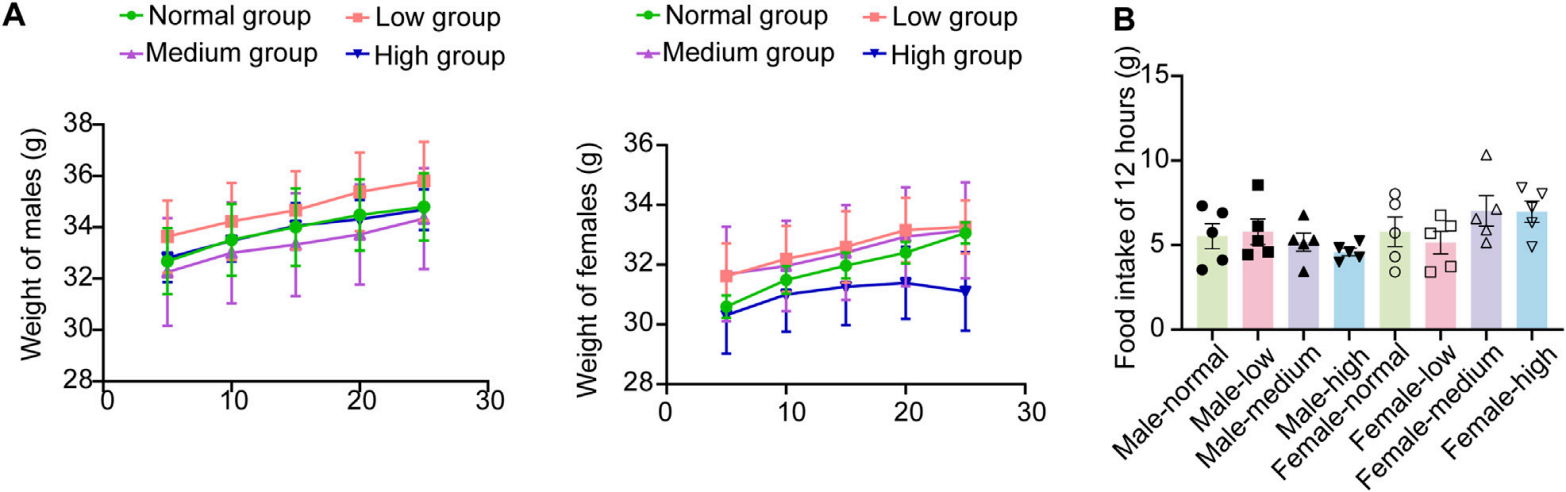
Weight and diet in subacute toxicology experiments. **(A)** Weight gain of male and female mice over 28 days. **(B)** Food intake of 12 h. Values were expressed as the mean ± SEM (*n* = 5).

During the 28-day subacute oral toxicity experiment, we closely monitored the mice in all groups. Overall, no significant abnormalities were observed in their fur, behavior, or respiration. Transient gastrointestinal disturbances were observed on day 5, with 4/10 high-dose and 3/10 mid-dose animals exhibiting soft feces. On day 19, one male mouse in the high group demonstrated compulsive grooming behavior, potentially related to minor gavage-induced irritation. Significantly, all aberrant symptoms resolved spontaneously within 24 h without residual effects. Comprehensive behavioral assessments revealed no persistent pathological alterations (detailed in Table 4). The results indicated no significant treatment effects on weekly body weight trajectories (Figure 3A, *P* > 0.05) or 12-h food intake (Figure 3B, *P* > 0.05). The basal metabolism remained intact even at the tested dose of 4,000 mg/kg, these findings support the safety profile of baicalin within the evaluated dosage range.

**TABLE 4:** General behavior of subacute toxicity studies.

Parameters	♂ (*n* = 5)				♀ (*n* = 5)			
	Normal	Low	Medium	High	Normal	Low	Medium	High
Appearance signs	0/5	0/5	0/5	0/5	0/5	0/5	0/5	0/5
Behavioral activities	0/5	0/5	0/5	1/5	0/5	0/5	0/5	0/5
Stimulus response	0/5	0/5	0/5	0/5	0/5	0/5	0/5	0/5
Secretions/excretions	0/5	0/5	1/5	1/5	0/5	0/5	2/5	3/5
Death	0/5	0/5	0/5	0/5	0/5	0/5	0/5	0/5

### 3.3 Changes in behavioral parameters

#### 3.3.1 Behavioral correlates of depression

**FIGURE 4:**
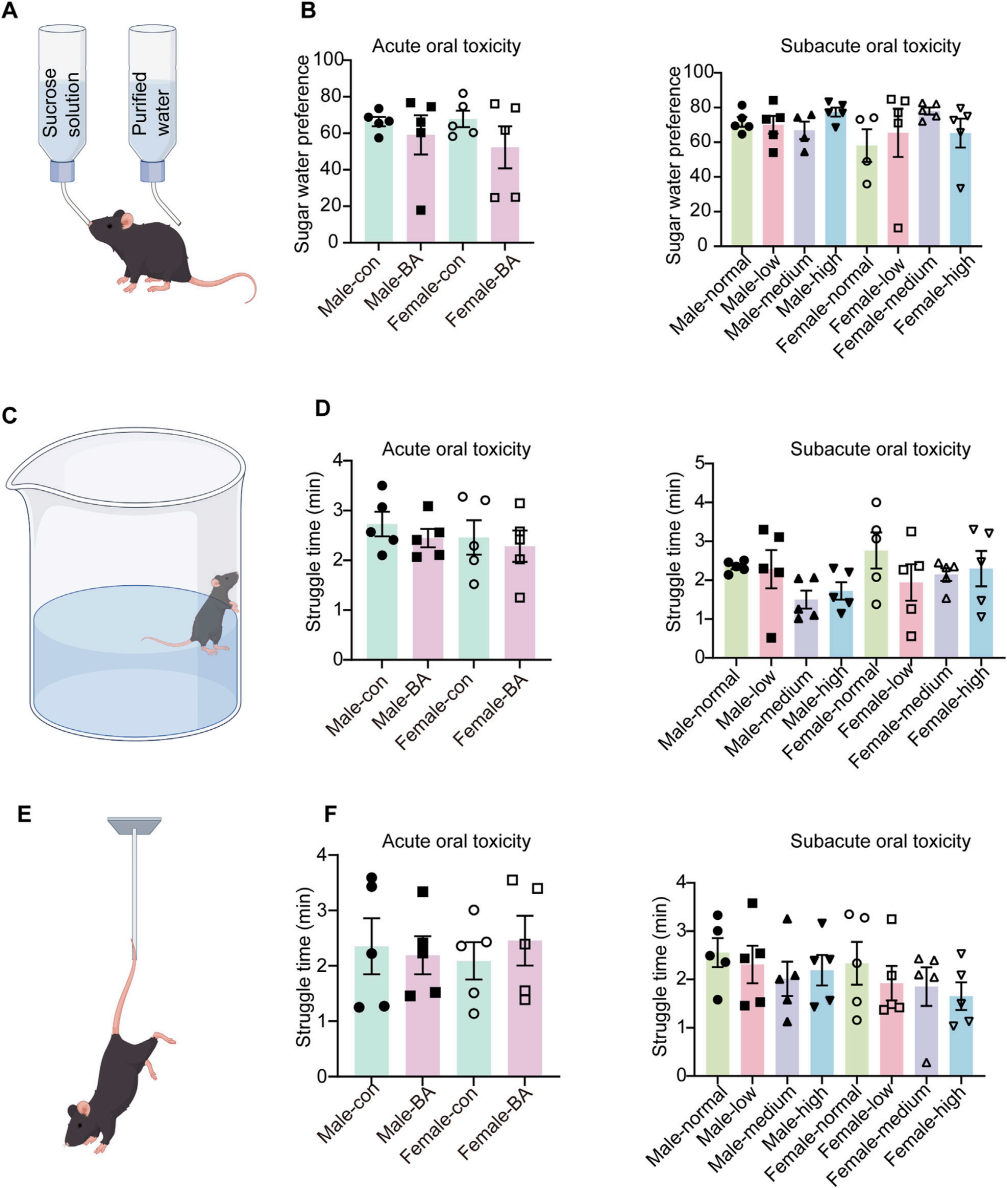
Alterations in depression-related behaviors of all groups. **(A)** Schematic diagram of the sucrose preference test. **(B)** Sugar water preference values of mice in each group (During the experiment, two mice were excluded from the SPT due to leaks in their water bottles). **(C)** Schematic diagram of the forced swim test. **(D)** Struggle time of mice in each group. **(E)** Schematic diagram of the tail suspension test. **(F)** Struggle time of mice in each group. Values were expressed as the mean ± SEM (*n* = 5).

To investigate the potential behavioral impacts of prolonged drug administration, this study integrated behavioral data from both acute and subacute toxicity. Whether in the acute or subacute toxicity stages, the sucrose preference ratios of mice in all groups did not have significant differences (Figures 4A,B, *P* > 0.05). During the experiment, two mice were excluded from the SPT due to leaks in their water bottles. The findings suggested that extended administration did not significantly impact the fundamental emotional state of the mice, as evidenced by the stability in their perception and preference for pleasurable stimuli.

Similarly, there were no significant differences in struggle time among mice during FST and TST (Figures 4B,C–E, *P* > 0.05). This phenomenon indicated the preserved behavioral despair resistance and psychological resilience following extended pharmacological exposure.

#### 3.3.2 Anxiety and compulsive behavior profiling

**FIGURE 5:**
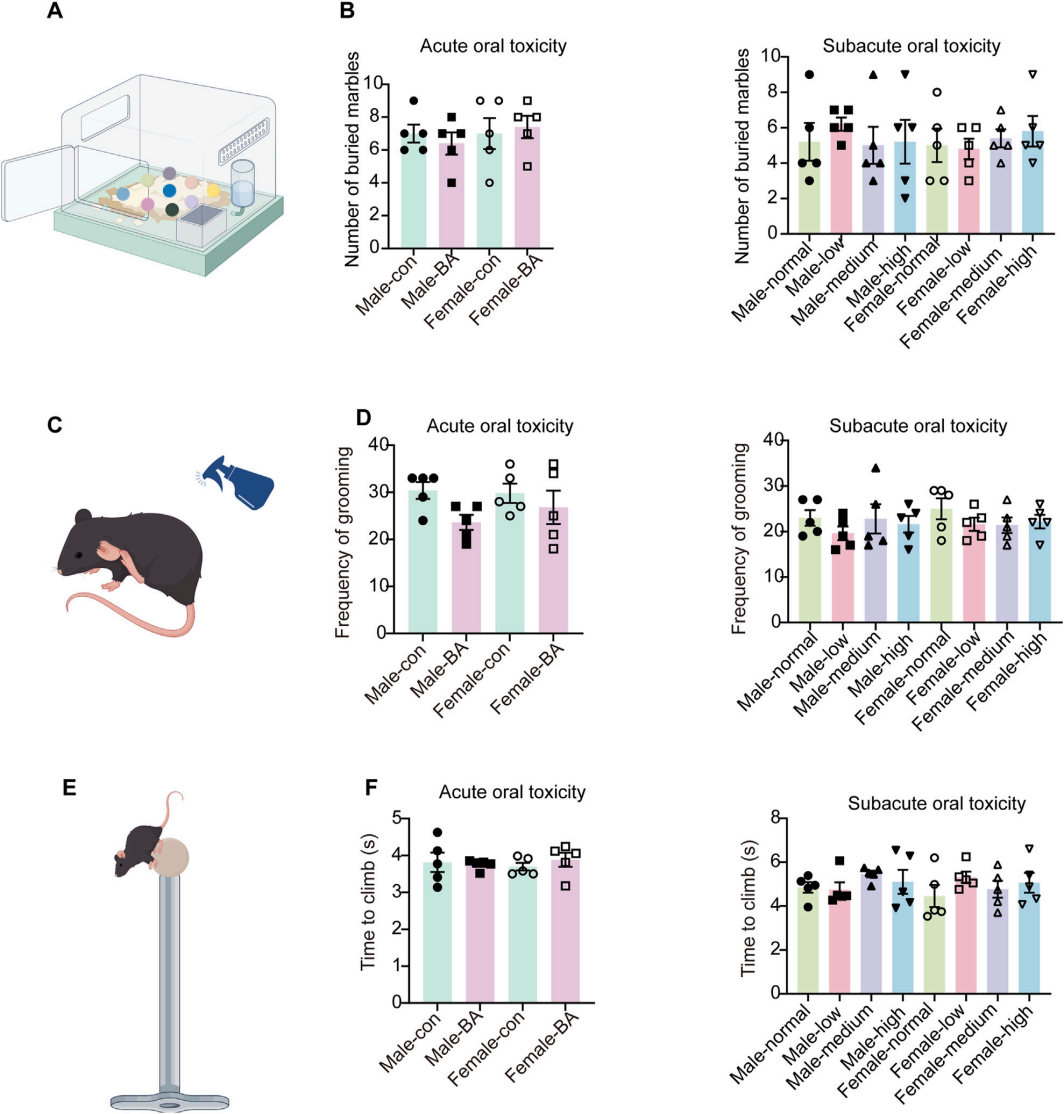
Anxiety and compulsive behavior profiling of all groups. **(A)** Schematic diagram of the marble-burying test. **(B)** Number of marbles buried in each group. **(C)** Schematic diagram of the sucrose splash test. **(D)** Frequency of grooming in each group. **(E)** Schematic diagram of the pole test. **(F)** Total time taken to climb down in each group. Values were expressed as the mean ± SEM (*n* = 5).

MBT is widely recognized as a key indicator of compulsive-like behavior in rodents. No significant differences in marble-burying counts were observed across all groups in both acute and subacute toxicity phases (Figures 5A,B, *P* > 0.05). Comparable frequencies of self-grooming were observed across all groups in the SST (Figures 5C,D, *P* > 0.05). This suggested that acute and subacute administration did not evoke significant alterations in stress responses or self-grooming behavior. Motor function assessments via the pole test revealed no dose-dependent effects of baicalin (Figures 5E,F, *P* > 0.05). Baicalin did not alter compulsive behaviors, stress reactivity, or motor function in either acute or subacute phases. These findings collectively validate its dose-independent neurobehavioral safety.

#### 3.3.3 Cognitive and innate behavioral assessments

**FIGURE 6:**
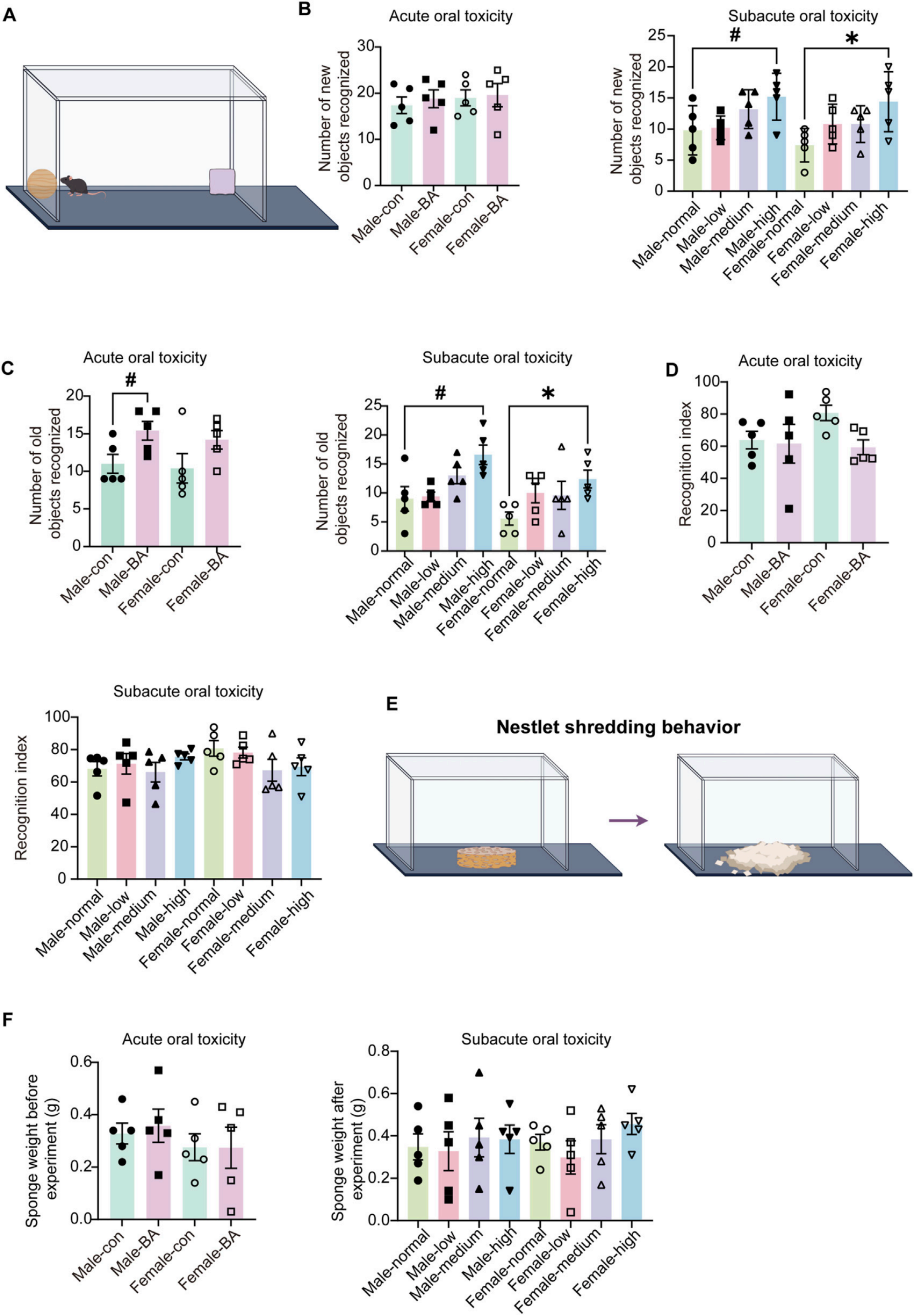
Cognitive and innate behavioral assessments of all groups. **(A)** Schematic diagram of the novel object recognition test. **(B)** Number of new objects recognized of mice in each group. **(C)** Number of old objects recognized of mice in each group. **(D)** Recognition index of mice in each group. **(E)** Schematic diagram of the nest shredding test. **(F)** Sponge weight in each group. Values were expressed as the mean ± SEM (*n* = 5). Compared to male normal group, #*P* < 0.05; Compared to female normal group, *P < 0.05.

To examine the effects of baicalin on the cognitive and instinctive behaviors of mice, this study employed the NOR and NST experiment. During the acute studies, no statistically significant difference emerged in the frequency of new objects (Figures 6A,B, *P* > 0.05). This finding tentatively indicated that short-term exposure to baicalin does not significantly alter in recognition-related behaviors. However, as the experiment progressed into the subacute toxicological studies, there was a marked increase in the frequency of recognition of both old and new objects (Figure 6C, *P* < 0.05). This observation suggested that prolonged exposure to baicalin may enhance the responsiveness of mice to novel stimuli at the behavioral level, which potentially indicated an increase in exploratory behavior. Nonetheless, it was important to note that further analysis of the recognition indices revealed no significant differences in recognition indices during either the acute or subacute toxicological phases (Figure 6D, *P* > 0.05).

NST demonstrated no significant changes in nest material disruption weights among all groups (Figures 6E,F, *P* > 0.05). These findings collectively demonstrated that baicalin does not impair cognitive processes or instinctual behaviors within the tested dose range, that supported safety profile of baicalin for chronic use.

### 3.4 Evaluation of organ coefficients

In order to gain a deeper understanding of the toxicological effects of baicalin, we employed organ-body weight ratios and organ-brain weight ratios as key indices. These parameters were selected for their potential to comprehensively reflect the impact of baicalin on the physiological state of experimental organisms at the organ level.

**FIGURE 7:**
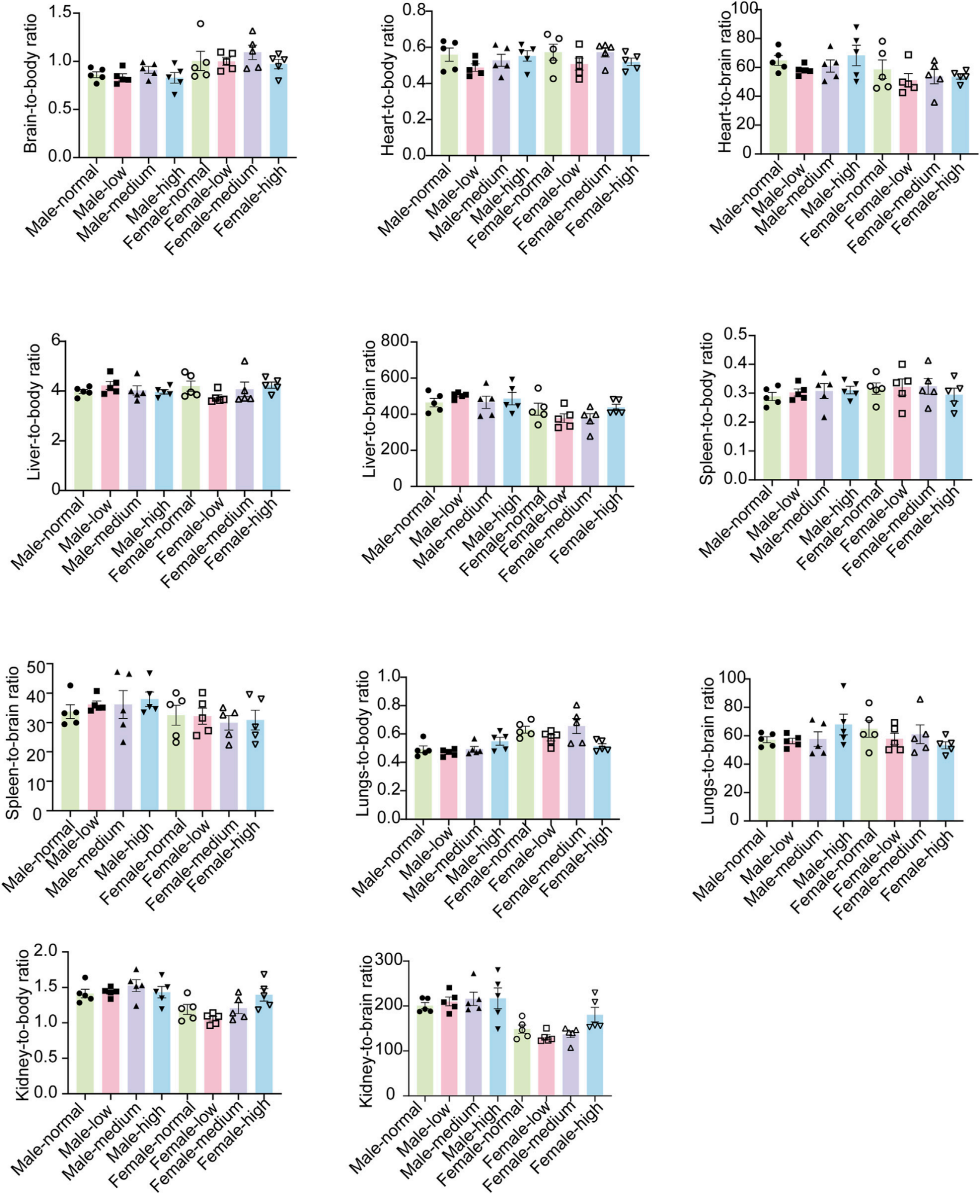
Organ-body weight ratios and the organ-brain weight ratios of mice in each group. Values were expressed as the mean ± SEM (*n* = 5).

No significant differences were observed among all groups in terms of either the organ-body weight ratios or the organ-brain weight ratios (Figure 7, *P* > 0.05). This finding implied that baicalin might not cause pronounced adverse effects in various organs. It provided valuable insights into the potential safety profile of baicalin during subacute exposure, suggested that its impact on organ-level physiological relationships might be minimal within the scope of our experimental design.

### 3.5 Routine blood assessment

The hematological analysis of mice demonstrated alterations in WBC and LYM%. Administration of a high dose of baicalin resulted in a statistically significant increase in WBC counts compared to the normal group (Table 5, *P* < 0.05). Male mice in the high-dose group showed a significant decrease in LYM% (*P* < 0.05), whereas female mice exhibited a non-significant trend (Table 5, *P* > 0.05). The hematological observations indicated that baicalin exerts dose-dependent immunomodulatory effects, as evidenced by leukocytosis and gender-specific lymphopenia. Collectively, these findings affirmed the safety profile of baicalin within the tested dosage range.

**TABLE 5:** Blood parameters for subacute toxicity studies.

Parameters	♂ (*n* = 5)				♀ (*n* = 5)			
	Normal	Low	Medium	High	Normal	Low	Medium	High
WBC (10^9^/L)	5.83 ± 0.35	5.76 ± 0.26	6.13 ± 0.85	8.99 ± 0.34#	5.12 ± 0.87	5.37 ± 0.38	5.35 ± 0.74	8.71 ± 1.04*
NEUT (%)	19.14 ± 1.20	18.78 ± 2.69	13.34 ± 2.78	16.92 ± 2.98	18.78 ± 2.69	19.58 ± 3.94	17.43 ± 1.28	17.61 ± 1.23
LYM (%)	80.28 ± 1.35	82.58 ± 2.45	83.16 ± 2.98	69.78 ± 4.80#	78.10 ± 3.94	81.44 ± 2.11	79.78 ± 1.75	82.52 ± 2.47
MONO (%)	0.52 ± 0.04	0.42 ± 0.02	0.60 ± 0.09	0.46 ± 0.05	0.44 ± 0.05	0.52 ± 0.06	0.48 ± 0.07	0.50 ± 0.04
EOS (10^9^/L)	0.02 ± 0.02	0.01 ± 0.00	0.01 ± 0.00	0.02 ± 0.01	0.02 ± 0.01	0.01 ± 0.01	0.02 ± 0.01	0.02 ± 0.01
BASO (10^9^/L)	0.02 ± 0.00	0.03 ± 0.01	0.01 ± 0.00	0.01 ± 0.00	0.01 ± 0.00	0.02 ± 0.01	0.02 ± 0.01	0.02 ± 0.01
RBC (10^12^/L)	10.19 ± 0.14	10.11 ± 0.28	10.34 ± 0.21	9.75 ± 0.33	10.07 ± 0.29	9.97 ± 0.04	10.03 ± 0.32	9.91 ± 0.28
HGB (g/L)	138.4 ± 2.50	138.4 ± 4.48	144.4 ± 2.82	142.2 ± 4.16	142.4 ± 4.03	141.40 ± 3.85	138.6 ± 0.93	137.2 ± 2.76
HTC (%)	52.84 ± 1.56	51.68 ± 1.62	54.06 ± 1.00	53.88 ± 1.53	53.22 ± 0.49	52.40 ± 1.24	54.04 ± 1.25	53.04 ± 0.80

Values were expressed as the mean ± SEM (*n* = 5). Compared to male normal group, #*P* < 0.05; Compared to male normal group, *P < 0.05.

### 3.6 Assessment of biochemical results

Liver function assessments demonstrated a dose-dependent decrease in ALP activity, with a particularly significant reduction observed in female mice within the high-dose group (Table 6, *P* < 0.05). This finding suggested a generalized modulation of ALP by baicalin. The observed decline may indicate altered hepatic metabolic activity, suggesting a pharmacological perturbation of liver physiology.

**TABLE 6:** Biochemical index for subacute toxicity studies.

Parameters	♂ (*n* = 5)				♀ (*n* = 5)			
	Normal	Low	Medium	High	Normal	Low	Medium	High
Liver function related parameters								
ALT (U/L)	31.12 ± 1.90	32.08 ± 2.27	33.86 ± 3.07	27.92 ± 2.06	33.94 ± 3.23	37.50 ± 2.07	33.64 ± 3.38	34.54 ± 3.78
AST (U/L)	127.96 ± 4.79	136.88 ± 7.04	144.84 ± 6.58	133.46 ± 2.85	138.03 ± 5.08	142.32 ± 6.69	144.21 ± 6.72	139.30 ± 6.47
ALP (U/L)	81.98 ± 5.25	75.98 ± 3.10	75.68 ± 1.84	54.88 ± 3.48#	81.05 ± 4.80	82.03 ± 5.47	74.86 ± 3.78	61.50 ± 2.51*
Renal function related parameters								
UA (μmol/L)	166.26 ± 4.52	160.54 ± 9.62	169.38 ± 7.34	175.24 ± 6.26	169.94 ± 7.45	167.31 ± 13.55	167.26 ± 12.49	166.46 ± 6.82
BUN (mmol/L)	8.66 ± 0.34	8.84 ± 0.37	9.70 ± 2.38	8.37 ± 1.23	7.83 ± 0.44	9.30 ± 1.84	10.17 ± 1.60	10.39 ± 1.88
SCr (μmol/L)	25.32 ± 0.23	23.32 ± 1.10	25.91 ± 1.56	27.87 ± 1.00	22.25 ± 2.33	24.64 ± 1.27	24.52 ± 1.74	27.05 ± 1.20*
Lipid related parameters								
TC (mmol/L)	4.28 ± 0.19	3.95 ± 0.18	4.02 ± 0.12	4.01 ± 0.19	4.14 ± 0.16	3.91 ± 0.10	4.02 ± 0.22	3.64 ± 0.27
TG (mmol/L)	1.17 ± 0.16	1.01 ± 0.23	1.21 ± 0.23	0.57 ± 0.16#	1.23 ± 0.22	1.09 ± 0.20	1.12 ± 0.19	0.68 ± 0.21*
Protein related parameters								
ALB (g/L)	35.42 ± 0.66	33.42 ± 0.84	35.56 ± 0.76	34.60 ± 1.21	34.74 ± 1.46	35.71 ± 0.41	38.30 ± 0.84	34.54 ± 1.26
TP (g/L)	60.65 ± 0.90	57.88 ± 1.23	61.97 ± 0.62	59.74 ± 1.12	60.74 ± 1.77	60.65 ± 2.59	59.88 ± 1.32	64.02 ± 1.18
Glob (g/L)	24.20 ± 0.69	23.78 ± 1.24	25.24 ± 0.53	25.86 ± 1.04	22.16 ± 0.93	23.48 ± 0.87	25.04 ± 1.32	24.88 ± 1.24

Values were expressed as the mean ± SEM (*n* = 5). Compared to male normal group, ^#^ *P* < 0.05; Compared to male normal group, *P < 0.05.

Renal function analysis showed significantly elevated SCr levels in female mice of the high-dose group (Table 6, *P* < 0.05), a biomarker of glomerular filtration impairment, which may indicated potential nephrotoxicity. Lipid profile evaluations demonstrated a significant reduction in TG levels in the high-dose group (Table 6, *P* < 0.05), consistent with baicalin’s putative lipid-lowering effects.

These biochemical findings showed that baicalin affected liver, kidney, and lipid metabolism. Reduced ALP and TG levels suggested beneficial metabolic effects, but increased Scr raised concerns about kidney safety.

### 3.7 Organ-specific histomorphometric analyses

**FIGURE 8:**
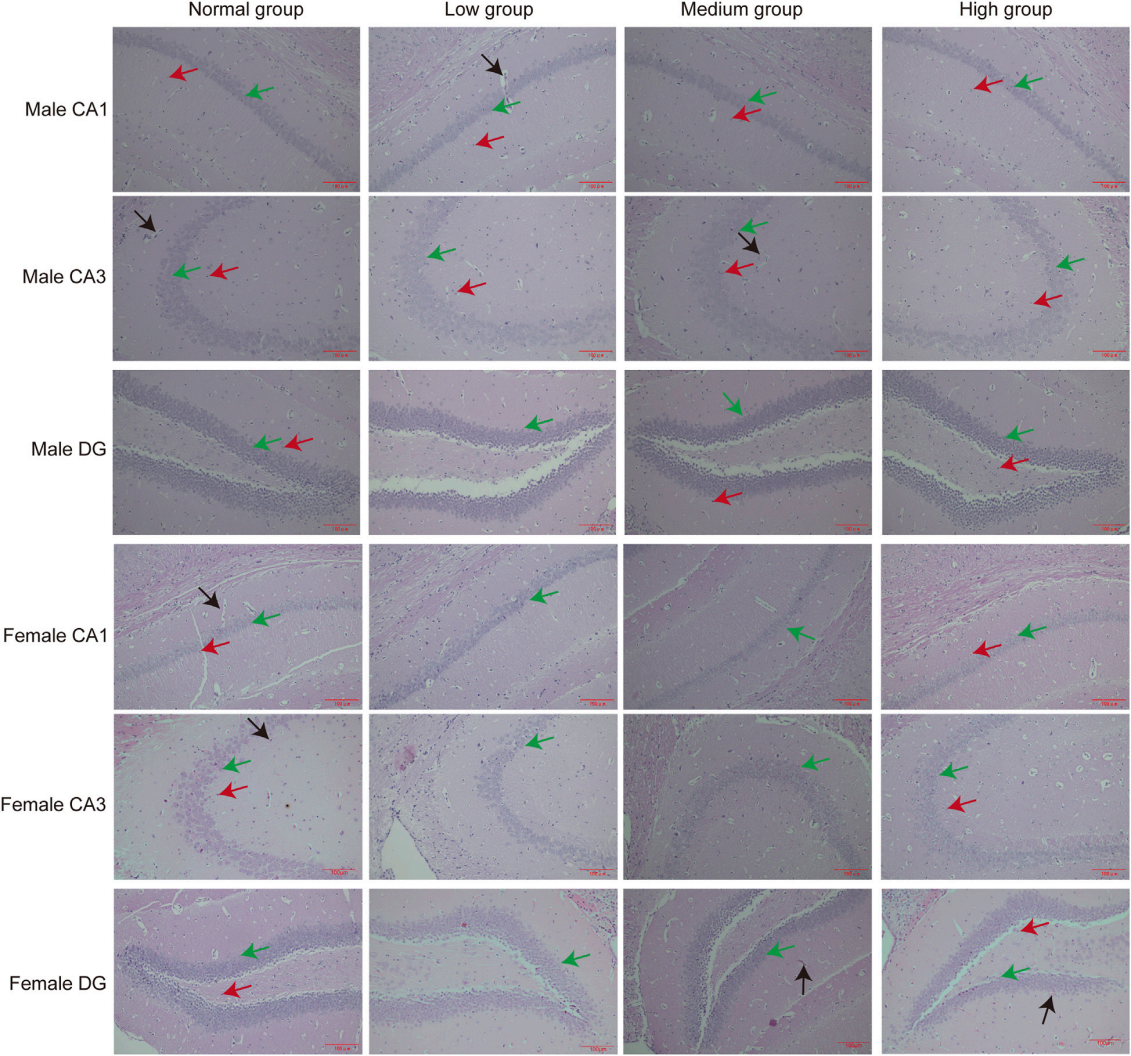
Pathologic results of hippocampal sections of mice in each group. Green arrows point to neuronal cells, red arrows point to glial cells, black arrows point to capillaries (*n* = 3).

To assess the toxicity profile of chronic baicalin exposure, we performed organ-specific histomorphometric analyses on hippocampal, hepatic and renal tissues. Considering the susceptibility of the hippocampus to neurotoxic damage, we conducted a systematic evaluation of its subregional integrity following prolonged exposure to high dose. Remarkably, our findings revealed no pathological changes in the CA1, CA3, or dentate gyrus (DG) neurons or glial cells. Histological analyses corroborated these findings, demonstrating preserved hippocampal cytoarchitecture and neuronal morphology (Figure 8).

**FIGURE 9:**
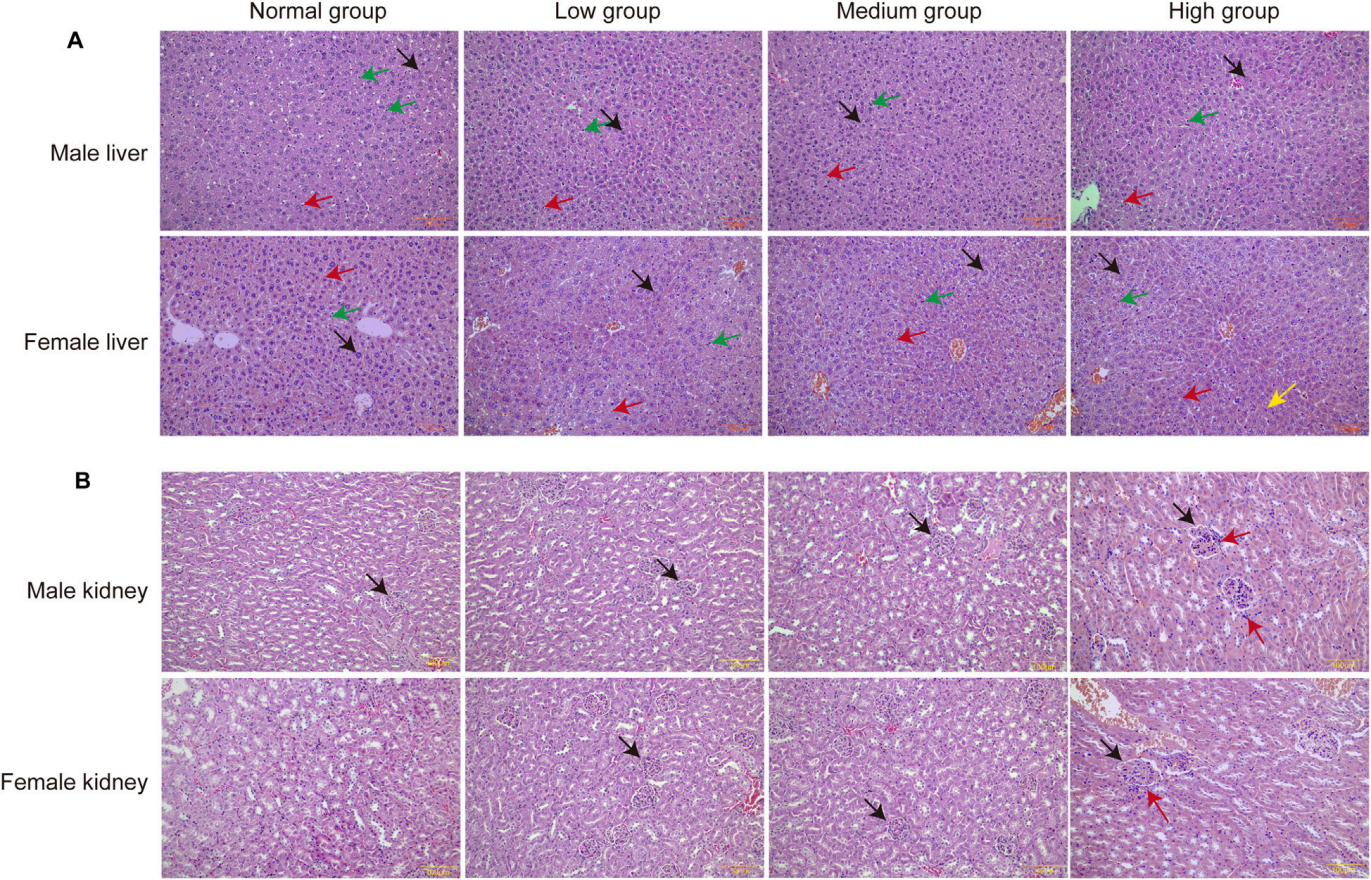
Pathologic results of liver and kidney sections of mice in each group. **(A)** Pathologic section of the liver. Green arrows point to Kupffer cells, red arrows point to endothelial cells, black arrows point to hepatocytes. **(B)** Pathologic section of the kidney. Black arrows point to glomeruli and red arrows point to inflammatory cell infiltration (*n* = 3).

In the morphological examination of liver histopathological sections, our analysis concentrated on alterations in hepatic sinusoidal endothelial cells (HSECs), Kupffer cells, and hepatocytes. The findings indicated that the group receiving a high-dose baicalin intervention exhibited pathological changes, characterized by hepatocytes displaying pyknosis and acidophilic chromatin condensation, which were indicative of an early metabolic stress state (Figure 9A).

Upon examination of the pathological sections of the kidney, it was observed that the glomeruli remained structurally intact with well-defined borders. However, a slight infiltration of inflammatory cells was noted surrounding the glomeruli following 28 days of exposure to a high dose of baicalin. This finding suggested that administration of baicalin at a dosage of 4,000 mg/kg may induce mild inflammation in the renal tissue (Figure 9B). Notably, these dose-dependent histopathological changed lacked progression to overt necrosis or fibrosis, aligning with transient SCr elevations.

## 4 Discussion

**FIGURE 10:**
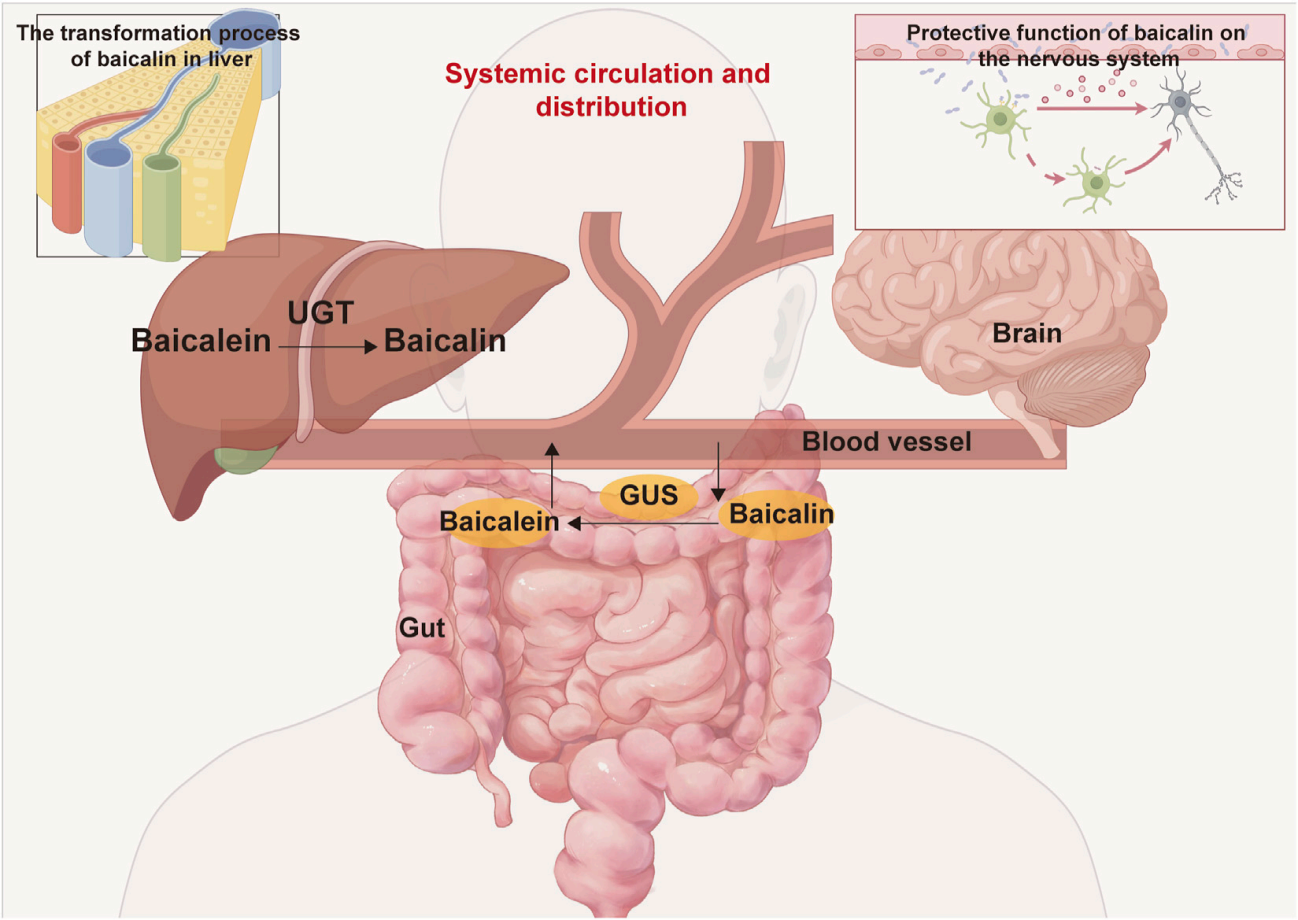
The metabolism and distribution of baicalin and baicalein within the body involve several key processes. Following oral administration, baicalin is transported to the intestine, where is enzymatically converted into baicalein by β-glucuronidase (GUS). Subsequently, the absorbed baicalein undergoes conversion back into baicalin via UDP-glucuronosyltransferase (UGT) in the liver, entering the enterohepatic circulation. Throughout this metabolic pathway, baicalin is capable of being distributed to various organs, including the brain, liver, gut, and others.

Baicalin, a flavonoid compound characterized by broad-spectrum biological activities, exhibits reduced membrane permeability due to its glycosylated structure, consequently attenuating its cytotoxicity (Chen et al., 2022; Froldi et al., 2022). The remarkable tolerance observed at 4,000 mg/kg is attributed to its unique pharmacokinetic characteristics, particularly its limited oral bioavailability. The extremely low aqueous solubility of baicalin (merely 16.82 μg/mL) restricts its dissolution rate and extent in the gastrointestinal tract (Li W. et al., 2018). Furthermore, its suboptimal lipid-water partition coefficient impedes passive transmembrane diffusion (Akao et al., 2000). Compounded by weak mucosal permeability, these factors collectively contribute to its poor oral bioavailability (Chen et al., 2022). The pharmacokinetic characteristics of baicalin are primarily reflected in carrier-mediated transport, enzyme-regulated metabolism, and enterohepatic circulation. Its intestinal absorption mainly relies on active transport mediated by specific transporters such as organic anion-transporting polypeptides (OATPs), rather than solely relying on passive diffusion. This transport mechanism renders its absorption susceptible to competitive inhibition by other drugs or compounds, thereby reducing bioavailability (Feng et al., 2019; Huang et al., 2019). Baicalin metabolism primarily depends on UDP-glucuronosyltransferases (UGTs). UGTs predominate in glucuronidation reactions (phase Ⅱ metabolism), generating water-soluble metabolites (e.g., baicalein glucuronide) to facilitate excretion (Yang et al., 2017; Wang R. et al., 2024). Furthermore, following biliary excretion, baicalin is hydrolyzed into aglycones (e.g., baicalein) by intestinal hydrolases (Xing et al., 2005). These aglycones are then reabsorbed into the circulation, thereby forming enterohepatic recycling (Figure 10) (Chen et al., 2014; Zhang et al., 2018). The absence of mortality and significant histopathological alterations at the 4,000 mg/kg aligns with OECD Guideline 420 criteria. Notably, transient fecal looseness in a few animals resolved spontaneously within 24 h, suggesting self-limiting gastrointestinal adaptation rather than systemic toxicity.

Sucrose preference indices were maintained, and struggle time in FST/TST remained unchanged, indicating that baicalin did not induce emotional dysregulation. While subacute treatment increased exploratory behavior towards novel stimuli, the intact object discrimination indicated preserved recognition memory, which aligns with serotonergic enhancement rather than cognitive impairment (Mustafa et al., 2025). These robust negative findings addressed critical regulatory concerns about phytochemical CNS liabilities, positioning baicalin as a preferential candidate for neuropsychiatric applications where synthetic antidepressants exhibit narrow therapeutic indices (e.g., SSRI-induced suicidal ideation).

Treatment did not significantly affect organ coefficients in male or female subjects, ruling out major structural adaptations like atrophy or compensatory hypertrophy. But the results of the blood test were the focus of our attention. Male mice treated with high-dose baicalin showed a statistically significant reduction in LYM%, whereas female mice exhibited no significant changes. This sex-based discrepancy may be attributed to baicalin-induced increases in estrogen and progesterone levels, with estrogen known for its immunomodulatory properties in females (Li et al., 2023). Estrogen potentially mitigates baicalin-induced immunosuppression by inhibiting pro-inflammatory cytokines or preserving lymphoid tissues (Al-Kuraishy et al., 2021; Fu et al., 2023; Li et al., 2023). Consequently, the endogenous hormonal environment in female mice helps maintain stable LYM% levels even under high-dose baicalin exposure, thereby preventing significant alterations. In contrast, male mice, lacking estrogen-mediated protective mechanisms, exhibited increased susceptibility to baicalin’s immunosuppressive effects, resulting in a marked decline in LYM%. Notably, numerous studies utilizing male mouse models, such as C57BL/6J or ICR strains, have documented baicalin-induced immunological changes, whereas comparative studies involving female mice remain limited (Wei et al., 2025).

Histopathological evaluations of the high-dose baicalin group showed that hepatocytes displayed pyknosis and acidophilic chromatin condensation, which were indicative of an early metabolic stress state. However, these changes failed to progress to overt necrosis or fibrosis. Biochemically, a dose-dependent decrease in ALP activity was observed, particularly significant in female mice of the high-dose group (Kimura et al., 2018). Studies have demonstrated that once the causative agent is eliminated, the liver can restore normal cellular morphology and enzyme activities (Porukala and Vinod, 2022; Xu et al., 2022). This is based on the liver’s well-known regenerative capacity. Regarding the kidneys, in the high-dose baicalin group, a slight infiltration of inflammatory cells was noted surrounding the glomeruli after 28 days of exposure. Elevated SCr levels were observed in female mice of the high-dose group. Similar to the liver, in related toxicology studies on compounds causing mild renal inflammation and transient changes in renal function markers, when the exposure is terminated, the renal tissue has been shown to resolve the inflammation, and the renal function markers return to normal levels (Yang M. et al., 2019; Ou et al., 2021). In our study, while we lacked post-exposure follow-up to directly demonstrate reversibility in the present experimental design, the absence of progression to more severe pathological states—such as glomerular damage, tubular necrosis, or chronic fibrosis—strongly indicates that the observed hepatic and renal changes are reversible.

Based on the translation of these findings into clinical practice, we suggest a murine no-observed-adverse-effect level (NOAEL) of 2,000 mg/kg/day, which corresponds to a human equivalent dose of approximately 162.16 mg/kg, as determined by the conversion factors presented in Table 7. This proposed dosage is consistent with clinical trials of baicalein, which have demonstrated safety at a dosage of 2,800 mg/day in humans, taking into account the lower bioavailability of baicalin (Li et al., 2014). To mitigate toxicity risks, nanoformulations (e.g., baicalin-loaded liposomes) could enhance bioavailability.

**TABLE 7:** Conversion of animal doses to human EquivalentDosesBased on body surface area.

Species	To convert animal dose in mg/kg to dose in mg/m^2^, Multiply by k_m_	To convert animal dose in mg/kg to HED^a^ in mg/kg, either	
		Divide animal dose by	Multiply animal dose by
Human	37	---	---
Child (20 kg)^b^	25	---	---
Mouse	3	12.3	0.08
Hamster	5	7.4	0.13
Rat	6	6.2	0.16
Ferret	7	5.3	0.19
Guinea pig	8	4.6	0.22
Rabbit	12	3.1	0.32
Dog	20	1.8	0.54
Primates			
Monkeys^c^	12	3.1	0.32
Marmoset	6	6.2	0.16
Squirrel monkey	7	5.3	0.19
Baboon	20	1.8	0.54
Micro-pig	27	1.4	0.73
Mini-pig	35	1.1	0.95

^a^ Assumes 60 kg human. For species not listed or for weights outside the standard ranges, HED, can be calculated from the following formula: HED, animal dose in mg/kg × (animal weight in kg/human weight in kg)^0.33^.

^b^ This k_m_ value is provided for reference only since healthy children will rarely be volunteers for phase l trials.

^c^ For example, cynomolgus, rhesus, and stumptail.

By integrating behavioral, biochemical, and pathological endpoints, this study not only redefines baicalin’s safety landscape but also establishes a translatable framework for evaluating natural product toxicity. As the quest for neuroprotective phytochemicals intensifies, our findings served as a timely reminder that “natural” does not equate to “harmless,” and rigorous safety pharmacology must accompany efficacy studies. It is noteworthy that due to interspecies metabolic disparities, these findings require validation in higher-order animal models. Long-term studies in neurodegenerative disease models are also essential to confirm their clinical translational value. Despite being a natural product, rigorous safety evaluation remains indispensable, and baicalin warrants further investigation as a low-risk candidate for neuroprotective applications.

## 5 Conclusion

This study showed that baicalin was safe for mice at dose up to 4,000 mg/kg, causing no deaths, major organ damage, or lasting neurobehavioral issues. Temporary liver and kidney changes were reversible, blood alterations indicated dose-related immune effects without reaching toxic levels. The NOAEL was 2,000 mg/kg suggesting its potential for long-term neurodegenerative treatments. Despite being natural, thorough safety checks was crucial, confirming baicalin as a low-risk neuroprotective option.

## Data Availability

The original contributions presented in the study are included in the article/supplementary material, further inquiries can be directed to the corresponding authors.
